# Association between contraceptive implant knowledge and intent with implant uptake among postpartum Malawian women: a prospective cohort study

**DOI:** 10.1186/s40834-016-0026-1

**Published:** 2016-08-10

**Authors:** Jennifer H. Tang, Dawn M. Kopp, Gretchen S. Stuart, Michele O’Shea, Christopher C. Stanley, Mina C. Hosseinipour, William C. Miller, Mwawi Mwale, Stephen Kaliti, Phylos Bonongwe, Nora E. Rosenberg

**Affiliations:** 1 UNC Project-Malawi, 100 Mzimba Road, Private Bag A-104, Lilongwe, Malawi; 2 Department of Obstetrics & Gynecology, University of North Carolina, 101 Manning Drive, CB #7570, Chapel Hill, NC 27599-7570 USA; 3 Department of Obstetrics & Gynaecology, Malawi College of Medicine, Mahatma Gandhi Road, Private Bag 360, Blantyre, Malawi; 4 Department of Obstetrics & Gynecology, Northwestern University, 250 East Superior Street, Suite 03-2303, Chicago, IL 60611 USA; 5 Department of Medicine, University of North Carolina, 130 Mason Farm Road, CB #7030, Chapel Hill, NC 27599-7030 USA; 6 Department of Epidemiology, University of North Carolina, 135 Dauer Drive, CB #7435, Chapel Hill, NC 27599-7435 USA; 7 Bwaila Hospital, Old Malangalanga Road, Lilongwe, Malawi; 8 UNC Institute for Global Health and Infectious Diseases, 130 Mason Farm Road, CB #7030, Chapel Hill, NC 27599-7030 USA

**Keywords:** Family planning, Long-acting reversible contraception, Postpartum, Africa, Subdermal implants, Intrauterine contraception

## Abstract

**Background:**

Long-acting reversible contraception (LARC) can assist women with birth spacing and reduce unintended pregnancies. Sub-Saharan Africa has low uptake of the two available methods of LARC, the subdermal implant and intrauterine contraception (IUC). Our primary objectives were to: 1) calculate the incidence of LARC use among postpartum Malawian women, and 2) assess if LARC knowledge and intent to use LARC were associated with LARC uptake.

**Methods:**

This study was a prospective cohort study of 634 postpartum women who were recruited from the postpartum ward of Bwaila Hospital in Lilongwe, Malawi. Study participants completed a baseline survey in the postpartum ward. Follow-up telephone surveys about contraceptive use were conducted at 3, 6, and 12 months postpartum. Cox proportional hazards regression analysis was performed to evaluate if implant knowledge and intent to use implant were associated with implant uptake.

**Results:**

One hundred thirty-seven implant and 10 IUC placements were reported over 12 months of follow-up; given the low rate of IUC uptake, further analysis was only done for implant uptake. The incidence rate for implant uptake was 35.6 per 100 person-years (95 % CI 30.0, 42.2). Correct implant knowledge (adjusted HR = 1.69; 95 % CI 1.06, 2.68) and intent to use implant (adjusted HR 1.95; 95 % CI 1.28, 2.98) were both associated with implant uptake.

**Conclusions:**

More women reported implant use than IUC use in our study. Correct implant knowledge and intent to use implant were both associated with implant uptake, with a stronger association for intent. Interventions to increase LARC uptake should focus on improving LARC knowledge and removing barriers to LARC.

**Trial registration:**

Clinical Trial Registration #: NCT01893021

## Background

Sub-Saharan Africa has high rates of unintended pregnancy, which contributes to adverse maternal, perinatal, and infant outcomes [1–3]. Subdermal implants and intrauterine contraception (IUC) are the most effective reversible contraceptives and are known as “long-acting reversible contraception” (LARC) [4, 5]. However, they are used by less than 3 % of reproductive-aged women (which includes postpartum women) in sub-Saharan Africa, including in Malawi [6, 7]. In Malawi, where 45 % of pregnancies are unintended, a recent study found that 97 % of postpartum women did not desire another pregnancy in the 2 years after delivery [7, 8]. Increasing LARC uptake among postpartum women may help to reduce both unintended pregnancies and lengthen interpregnancy intervals [9–11].

Understanding the modifiable characteristics of women who initiate and continue LARC in the first year postpartum can help guide strategies to increase LARC uptake. Low rates of LARC uptake may stem from lack of interest, accurate knowledge, [12, 13] peer and partner support, or access [14, 15]. For our study, we chose to focus on the potential associations between LARC knowledge and intent to use LARC with LARC uptake. Correct LARC knowledge may increase intent to use LARC, which can then lead to increased LARC uptake if other barriers to LARC do not exist. Therefore, our primary objectives were to: 1) calculate the incidence rate of LARC use among postpartum Malawian women, and 2) assess if LARC knowledge and intent to use LARC were associated with LARC uptake. Our secondary objectives were to describe barriers to LARC uptake and reasons for discontinuation among LARC users.

## Methods

### Study setting and population

We conducted a prospective cohort study of postpartum Malawian women who delivered between May-September 2013 [8]. Approval was obtained from the University of North Carolina School of Medicine Institutional Review Board and the Malawi National Health Sciences Research Committee.

Women were recruited from the postnatal unit of Bwaila Hospital, a district hospital in Lilongwe, Malawi, with over 15,000 deliveries per year. Antenatal counseling on family planning is not provided in a standardized fashion in the antenatal clinics due to the frequent rotation of providers in the clinic and its high volume of clients, but may be discussed if a client or provider chooses to initiate a conversation about family planning. Immediate postpartum contraception is not offered at Bwaila. Two methods of LARC are available in Malawi: the subdermal implant (both the etonogestrel and levonorgestrel implants) and the copper intrauterine device.

### Study design

Women were recruited and enrolled from the postnatal ward. The six inclusion criteria were: current admission to the postpartum ward at Bwaila Hospital, age 18–45 years, live birth at greater than 28 weeks gestation, fluency in English or Chichewa (the local language), access to a working phone, and willingness to be contacted by phone for up to 1 year postpartum. Eligible women provided informed consent and completed a 30-min baseline survey focused on demographics, family planning preferences, and knowledge of LARC.

Follow-up surveys were conducted by phone at approximately 3, 6, and 12 months after delivery and focused on contraceptive uptake and continuation. Participants also received a reminder phone call about the upcoming 12-month survey 9 months after delivery, but no survey was administered. Women who missed a survey were not contacted for subsequent surveys, although one participant who had not completed either the 3- or 6-month survey was inadvertently called for the 12-month survey and completed it. For women who were eligible to complete the 12-month survey, we attempted to contact these women (up to 17 months postpartum) to complete this final survey until all study participant contact ended in October 2014.

### Variables

Timing of LARC uptake and continuation was based on participants’ reported date of initiation or discontinuation of these methods. Participants were considered to have correct knowledge about implant or IUC efficacy if they answered that the LARC method was more effective at preventing pregnancy than injectables, which is known by 95 % of women in Malawi [7]. Participants were also asked, “Do you think the implant/IUC is a safe or unsafe form of family planning?” Those who responded “safe” were determined to have correct knowledge about LARC safety. Women were categorized as having correct implant knowledge if they answered both the implant efficacy and safety question correctly; they were categorized as having correct IUC knowledge if they answered both the IUC efficacy and safety questions correctly. Intention to use IUC or implant was determined from the participant’s response at the baseline survey when asked about the contraceptive methods she was planning to use in the first year after delivery. At each follow-up call, women who were not using an implant or IUC were asked if they would like to be currently using either of these methods, and if so, their reasons for non-uptake. Similarly, women who had discontinued LARC were asked about their reasons for discontinuation.

### Sample Size

We determined that a convenience sample of approximately 630 postpartum women (210 HIV-infected women, 420 HIV-uninfected women) would be necessary for our primary study objective, which was to compare baseline LARC knowledge between HIV-infected and uninfected women (at a 1:2 ratio). The results of the primary study objective have already been published [8]. Since most of the women at the recruitment site were HIV-uninfected, to maintain the 1:2 ratio we capped the number of uninfected women that could be enrolled each week, based on the number of HIV-infected women that were enrolled that week.

### Statistical analysis

All data were double entered into a REDCap database, cleaned, merged, exported, and analyzed using Stata Version 13.0 (StataCorp, College Station, TX). Pearson’s χ^2^ tests were used to compare distributions of categorical variables. For incidence rate calculations and time to event analyses, the baseline survey was the origin for survival time and participants were followed until either time of implant uptake or last completed survey date. We calculated incidence rates and 95 % confidence intervals (CIs) of implant uptake for each baseline variable for 100-person-years of exposure time using the Stptime procedure in Stata. The relationship between correct implant knowledge and intent to use implant with time of implant uptake was evaluated using unadjusted and adjusted Cox proportional hazards regression models. We used directed acyclic graphs to identify potential confounders for implant knowledge and intent to use implant to include in the final adjusted models. We estimated time to implant uptake by constructing Kaplan-Meier survival curves and used the non-parametric log-rank test to compare implant uptake by our two predetermined variables, correct implant knowledge and intent to use implant.

## Results

### Study population

**Fig. 1:**
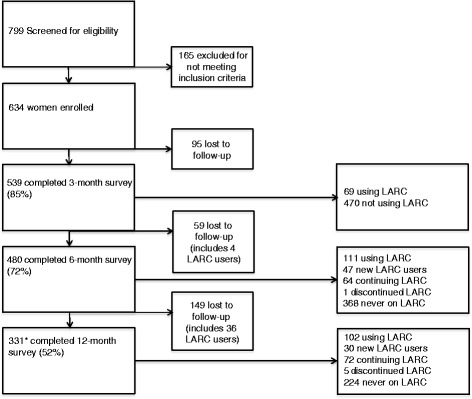
Implant or intrauterine contraceptive (IUC) use among postpartum Malawian women up to 12 months after delivery. *One woman completed the 12-month survey, but not the 3- or 6-month survey

We screened 799 women and 634 enrolled (Fig. 1). The most common reason for non-enrollment was not having access to a working phone (15 %). Follow-up data were collected from 539 (85.0 %), 480 (75.7 %), and 331 (52.2 %) women at 3, 6, and 12 months, respectively. The median age was 25 years (IQR 21, 29) and the mean parity was 2 (IQR 1, 3).

Of the 634 women who enrolled, 613 (96.7 %) responded to both survey questions about correct implant knowledge and were included in the implant knowledge analyses (Table 1). More women had correct knowledge about the contraceptive implant than had correct knowledge about IUC. Eighty-four percent correctly identified the implant as safe and 79.8 % correctly said it was more effective than injectables, with 72.9 % answering both implant knowledge questions correctly. In contrast, 55.8 % of women correctly identified IUC as safe, 46.5 % correctly identified IUC as more effective, and 38.0 % answered both IUC knowledge questions correctly. In addition, more women desired to use implant (67.4 %) than desired to use IUC (19.7 %) at the time of the baseline survey. More women also reported a friend using the implant (76.7 %) than a friend using IUC (36.4 %). Women who responded with correct implant knowledge were more likely to have correct IUC knowledge (*p* < 0.01), desire an implant (*p* < 0.01), and have a friend who had used the implant (*p* = 0.01) (Table 1).

**Table 1:** Characteristics of women with and without correct implant knowledge at baseline

	Correct Implant Knowledge (*n* = 462)	Incorrect Implant Knowledge (*n* = 151)	*p*-value
Age, n (%)			
18–24	215 (46.5)	70 (46.4)	0.75
25–34	208 (45.0)	71 (47.0)	
≥35	39 (8.4)	10 (6.6)	
Relationship status, n (%)			
Married	440 (95.2)	138 (91.4)	0.08
Unmarried	22 (4.8)	13 (8.6)	
Education, n (%)			
None or some primary	127 (27.5)	46 (30.5)	0.29
Primary/some secondary	217 (47.0)	60 (39.7)	
Secondary and beyond	118 (25.5)	45 (29.8)	
Trouble with food, clothing, or medications, n (%)^a^			
Yes	254 (54.9)	91 (60.3)	0.28
No	206 (44.6)	60 (39.7)	
Living children, n (%)			
1	171 (37.0)	60 (39.7)	0.75
2–3	206 (44.6)	62 (41.1)	
≥4	85 (18.4)	29 (19.2)	
Desire any more children, n (%)^a^			
Yes	286 (61.9)	88 (58.3)	0.72
No	167 (36.1)	59 (39.1)	
Don’t know	7 (1.5)	3 (2.0)	
Most recent pregnancy intention, n (%)			
Intended	270 (58.4)	89 (58.9)	0.21
Unintended	192 (41.6)	61 (40.4)	
Don’t know	0 (0.0)	1 (0.7)	
HIV status, n (%)			
Infected	157 (34.0)	49 (32.5)	0.73
Uninfected	305 (66.0)	102 (67.6)	
Knowledge about IUC Safety and Efficacy, n (%)^a^			
Correct	216 (46.8)	23 (15.2)	<0.01
Incorrect/Don’t Know	216 (46.8)	116 (76.8)	
Intent to use Implant, n (%)			
Yes	343 (74.2)	71 (47.0)	<0.01
No	119 (25.8)	80 (53.0)	
Intent to use IUC, n (%)			
Yes	88 (19.1)	32 (21.2)	0.56
No	374 (81.0)	119 (78.8)	
Have friend using the implant, n (%)^a^			
Yes	371 (80.3)	105 (69.5)	0.01
No	86 (18.6)	42 (27.8)	

*IUC* intrauterine contraception

^a^may not add up to 100 % due to missing data

A total of 146 participants reported LARC use during the follow-up period. LARC uptake was reported by 69 participants at the 3-month survey, another 47 participants at the 6-month survey, and an additional 30 participants at the 12-month survey (Fig. 1). One-hundred and thirty-seven implant and 10 IUC placements were reported; one participant who had an implant placed had it removed and had IUC placed instead. Of the implant users, 101 (73.7 %) reported that they received their implants from a government facility, whereas only 4 (40.0 %) IUC users received their method at a government facility. Due to the small numbers of IUC users, additional analyses were only performed on implant uptake. Four implant users did not give a placement date and were not included in time-to-event analysis.

Implant uptake varied by baseline intention to use the implant. Of the 137 women who received an implant, 30 (21.9 %) did not plan to use it during the baseline survey; 43 (31.4 %) reported a plan to use both the implant and another method; and 64 (46.7 %) reported a plan to use only the implant. In contrast, only 2 (20.0 %) of the 10 women who initiated the IUC reported a plan to use only IUC at baseline; 7 (70.0 %) did not report a plan to use it at all at baseline, and the remaining woman (10.0 %) reported a plan to use both IUC and another method.

Among the 331 women who reported that they wanted to use the implant at a follow-up survey but did not initiate it during the study, the most common reasons given for not initiating were “currently using another method” (52.0 %), “afraid of the side effects” (23.2 %), and “clinic did not have it or said to come back for another appointment” (18.4 %). The reasons given for not initiating IUC among the 59 women who reported that they wanted to use IUC but did not initiate it were similar: 42.4 % were “currently using another method”, 16.9 % were “afraid of side effects”, and 10.2 % reported that the “clinic did not have it or said to come back for another appointment”. In total, 357 women reported that they wanted to use the implant and/or IUC at a follow-up survey but were not using it. When asked what family planning method they were using instead, the majority (67.9 %) were using the contraceptive injection, 7.5 % were using oral contraceptives, 4.4 % were using breastfeeding, and 20.1 % were using no contraceptive method.

### Main analyses

The median time for implant placement was 3 months from delivery (IQR 2–5), which was the same as the time to IUC placement (3 months; IQR 2–5). One hundred and thirty-three implant uptakes were seen in 373.5 person-years of follow-up, at an incidence rate of 35.6 per 100 person-years (95 % confidence interval 30.0, 42.2) (Table 2). Women with correct baseline implant knowledge had an implant incidence rate of 39.1 per 100 person-years [95 % CI 32.3, 47.4], whereas women with incorrect implant knowledge had an implant incidence rate of 24.0 per 100 person-years (95 % CI 16.0, 36.2), which was not statistically significant. However, women who had an intent to use the implant at baseline had a statistically significant higher implant incident rate (42.7 per 100 person-years; 95 % CI 35.2, 51.8) than those who did not intend to use it (22.7 per 100 person-years; 95 % CI 15.9, 32.4). Those women who had both correct implant knowledge and an intent to use the implant at baseline had a significantly higher implant incident rate (44.8 per 100 person-years; 95 % CI 36.2, 55.3) than those women who did not have both (25.9 per 100 person-years; 95 % CI 19.5, 34.5).

**Table 2:** Incidence of Implant uptake by correct implant knowledge and covariates

	Implant Uptake Events (n)	Person-years	Incidence Rate (per 100 person-years)	95 % CI of IR
Overall	133	373.5	35.6	(30.0, 42.2)
Implant Knowledge				
Correct	105	268.3	39.1	(32.3, 47.4)
Incorrect	23	95.7	24.0	(16.0, 36.2)
Age categories				
18–24	63	165.5	38.1	(29.7, 48.7)
25–34	60	174.6	34.4	(26.7, 44.3)
≥35	10	33.4	30.0	(16.1, 55.7)
Relationship status				
Married	129	348.6	37.0	(31.1, 44.0)
Unmarried	4	24.9	16.1	(6.0, 42.8)
Education				
None or some primary	29	95.7	30.3	(21.1, 43.6)
Primary/some secondary	62	162.0	38.3	(29.8, 49.1)
Secondary and beyond	42	115.9	36.2	(26.8, 49.1)
Trouble with food, clothing, or medications				
Yes	69	194.8	35.4	(28.0, 44.8)
No	63	177.8	35.4	(27.7, 45.4)
Living children				
1	47	144.2	32.6	(24.5, 43.4)
2–3	67	160.6	41.7	(32.8, 53.0)
≥4	19	68.7	27.7	(17.6, 43.4)
Desire any more children				
Yes	82	233.9	35.1	(28.2, 43.5)
No	47	130.3	36.1	(27.1, 48.0)
Don’t know	3	8.0	37.4	(12.0, 115.8)
Most recent pregnancy intention				
Intended	85	224.8	37.8	(30.6, 46.8)
Unintended	48	148.4	32.3	(24.4, 42.9)
Don’t know	0	-	-	-
HIV status				
Infected	38	121.4	31.3	(22.8, 43.0)
Uninfected	95	252.1	37.7	(30.8, 46.1)
Intent to use Implant				
Yes	103	241.2	42.7	(35.2, 51.8)
No	30	132.3	22.7	(15.9, 32.4)
Have friend using the implant				
Yes	105	287.8	36.5	(30.1, 44.2)
No	26	79.1	32.8	(22.4, 48.3)
Both correct knowledge about implant and intent to use implant				
Yes	86	192.1	44.8	(36.2, 55.3)
No	47	181.4	25.9	(19.5, 34.5)

**Fig. 2:**
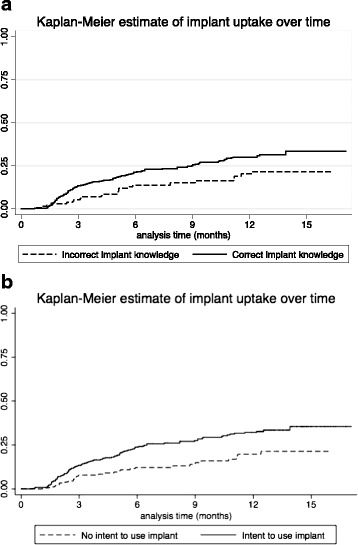
**a** Kaplan-Meier estimate of contraceptive implant uptake over time by correct versus incorrect implant knowledge. * *p* = 0.030 by log-rank test. **b** Kaplan-Meier estimate of contraceptive implant uptake over time by intent to use implant at baseline. *p = 0.002 by log-rank test

In the Kaplan-Meier survival analysis, women with correct implant knowledge had a shorter time to implant uptake than those with incorrect knowledge, as did those who intended to use an implant at baseline (Fig. 2). In unadjusted Cox proportional hazards regression modeling, correct implant knowledge was associated with higher implant uptake (unadjusted HR 1.63; 95 % CI 1.04, 2.55). This association remained significant (adjusted HR = 1.69; 95 % CI 1.06, 2.68) when adjusted for age, parity, education, having a friend using implant, and HIV status. Our Cox proportional hazards regression model for intent to use implant adjusted for age, parity, education, having a friend using the implant, HIV status, and having trouble obtaining food/clothing/medications. Both the unadjusted HR (1.88; 95 % CI 1.25, 2.82) and adjusted HR (1.95; 95 % CI 1.28, 2.98) for intent to use implant at baseline were statistically significant.

### Continuation analysis

Six women discontinued the implant; no women discontinued the IUC. Three women discontinued due to heavy bleeding. Other reasons given for discontinuation were abdominal pain or cramping (*n* = 2), arm pain (*n* = 1), headache (*n* = 1), lightheadedness (*n* = 1), weight loss (*n* = 1), and hypertension (*n* = 1). Half of these women discontinued the implant 3–6 months after placement (*n* = 3), and the rest discontinued between 6–12 months after placement. Due to the small number of women who reported discontinuation of implant in the study period, statistical analysis to examine the differences between women who continued or discontinued implant or IUC were not performed.

## Discussion

In this prospective cohort study of postpartum Malawian women, we found a substantial proportion of women initiating LARC with an implant uptake incidence rate of 35.6 per 100 person-years. We also found that contraceptive implants were utilized more than IUC. Women with correct baseline implant knowledge and who intended to use implant were more likely to initiate implant. The association between intent to use implant and implant uptake was stronger than the association between correct implant knowledge and implant uptake.

Interest, correct knowledge, and uptake were lower for IUC than for implant in our study population, which is consistent with other data from Malawi and sub-Saharan Africa. In the 2010 Malawi Demographic Health Survey, slightly more women between ages 15–49 years knew of the implant (77.6 %) than IUC (73.8 %) [7]. In that same survey, only had 1.7 % and 0.8 % of women had ever used the implant and IUC, respectively. More women were currently using implant (1.1 %) than IUC (0.2 %). Studies from Uganda and Ethiopia have also demonstrated lower knowledge about IUC than about implant or injectables [15, 16]. A Rwandan study of HIV-infected women followed for 9 months after delivery had a 10 times greater absolute implant uptake than IUC uptake [17].

Low IUC uptake in Malawi is likely secondary to both patient and provider misperceptions and barriers. A cross-sectional study of 281 HIV-infected women accessing care at an antiretroviral therapy clinic in Lilongwe found that 25.4 % feared IUC placement and 25.4 % had heard negative things about IUC [18]. However, the same clinic found that after they integrated family planning education and on-site IUC insertion, 165 (19 %) of the 859 women who initiated modern contraception during their integration time period had IUC inserted [19]. A recent mixed-methods study of 37 government family planning providers in Lilongwe District found that while providers had a generally favorable perception of IUC, only 11 (29.7 %) had received any training in IUC, 6 (16.2 %) had inserted IUC during their training, and 8 had ever-inserted IUC (21.6 %) [20]. Some women in our study who were interested in IUC stated that they were not using it because of clinic-related issues or because they were already using another method. The lack of experienced IUC providers and high workload have likely resulted in limited availability of IUC at government health centers, where they may opt to provide a less work-intensive method such as the injectable or pills.

Although implant uptake was higher than IUC uptake in our study, barriers to uptake for both LARC methods were reported by our participants, and the majority of women interested in LARC at baseline still had not received it by the time they completed the 12-month follow-up survey. Increasing knowledge of LARC and intent to use LARC alone will not increase LARC uptake if other barriers to LARC are not removed, which is probably why the associations between these two characteristics with LARC uptake were not very strong. The clinic barriers and women’s fears about LARC must also be addressed.

Other studies in sub-Saharan Africa have also shown an association between contraceptive knowledge and LARC use, although they used different measures for contraceptive knowledge [15]. In a cross-sectional study from South Africa, [21] contraceptive knowledge was assessed through questions about IUC safety among HIV-infected and breastfeeding women, side effects of IUC, duration of effectiveness, and return to fertility after removal. More than half of the participants in that study did not have correct IUC knowledge for most of these questions and no participants were currently using IUC. Characteristics such as age, marital status, education levels, and previous LARC use have been associated with LARC uptake in other sub-Saharan populations [22, 23]. We did not find these characteristics to be associated with LARC uptake in our population. Importantly, these studies did not include postpartum women.

Our study differs also from other studies in that we had a relatively high rate of postpartum implant uptake when compared to other studies [13, 21, 24]. Our high rate of implant uptake may be due to the design of this study. By asking women to complete a survey regarding implant and IUC knowledge and intentions, we may have increased LARC interest and uptake. In addition, by limiting our study population to women in an urban setting with access to a working telephone, we may have excluded women who have more geographic and socioeconomic barriers to receiving LARC and decreased the generalizability of our findings. A Hawthorne (observer) bias also may have increased LARC uptake in our study population as women anticipating a call asking about LARC use every 3 months may be more motivated to have LARC placed. Women may have been tempted to report LARC placement when this had not occurred, although we attempted to minimize this social desirability and information bias by asking for the exact date of LARC placement. However, no verification of LARC use with clinic records could be performed due to the study methodology.

In addition, since we did not retain all women in this study, we may be misestimating the proportion of women using and discontinuing LARC. To explore the potential effect of loss-to-follow-up, we compared the baseline characteristics between the 540 women who completed at least one follow-up survey with the 94 women who did not complete any follow-up surveys. Women who did not complete any follow-up surveys were less educated (*p* = 0.003) and less likely to have 2–3 living children (*p* = 0.015) than women who completed at least one follow-up survey, but were otherwise similar. Another limitation is that we used only two survey questions to categorize women as having correct implant knowledge; other studies that use a different definition of correct knowledge may result in different findings.

This prospective study is important because it is one of the few published studies to describe patterns of contraceptive uptake and discontinuation among postpartum women with 1 year of follow-up in sub-Saharan Africa. Other studies among postpartum women have only been able to assess contraceptive uptake at hospital discharge or within the first 3 months postpartum [24, 25]. We were able to describe baseline characteristics of women who used LARC over 1 year and the timing of use and discontinuation. In addition, the large sample size allowed us to do more in-depth analyses of factors associated with implant use.

Future studies should focus on further understanding the barriers to LARC uptake and implementing educational interventions to increase LARC knowledge. The optimal nature of and timing of educational interventions has not been established. Two studies have shown that immediate postpartum education can increase contraceptive use; one specifically showed an increased in IUC use [26, 27]. Another study from the U.S. showed increased LARC interest but not uptake with an immediate postpartum script [24]. One study in Zimbabwe found that contraceptive counseling during antenatal care increased IUC knowledge, but not uptake [28]. Educational campaigns through radio, [29] billboards, and social media [30] have shown to be effective at increasing contraceptive uptake and continuation in some studies.

Contraceptive knowledge can also be obtained outside of the healthcare setting. Our study demonstrated that having a friend with an implant was associated with correct implant knowledge, but not implant uptake. Studies of adolescents in Nigeria [31] and undergraduates in Tanzania [32] both found that the most common source of contraceptive information was from their friends. Therefore, social networks may play an important role in contraceptive knowledge and uptake and could be targeted as another intervention point.

## Conclusions

In summary, to increase LARC uptake in postpartum women, we need to focus on interventions to increase correct contraceptive knowledge, which may be one step towards increasing demand for and ultimately uptake of LARC. We also need to ensure that women who intend to use LARC are able to access it. Strategies to determine which types of educational interventions could be successful at increasing LARC knowledge, uptake, and continuation for postpartum women and enable them to reach their fertility goals.

## References

[1] Kassebaum NJ, Bertozzi-Villa A, Coggeshall MS (2014). Global, regional, and national levels and causes of maternal mortality during 1990-2013: a systematic analysis for the Global Burden of Disease Study 2013. Lancet.

[2] Mayer JP (1997). Unintended childbearing, maternal beliefs, and delay of prenatal care. Birth.

[3] Orr ST, Miller CA, James SA (2000). Unintended pregnancy and preterm birth. Paedr Perinat Epidemiol.

[4] Trussell J (2011). Contraceptive failure in the United States. Contraception.

[5] Winner B, Peipert JF, Zhao Q (2012). Effectiveness of long-acting reversible contraception. NEJM.

[6] United Nations, Department of Economic and Social Affairs, Population Division (2013). World contraceptive patterns. New York, New York, USA. POP/DB/CP/Rev2013.

[7] National Statistical Office (NSO) and ICF Macro. Malawi Demographic and Health Survey 2010. Zomba, Malawi, and Calverton, Maryland, USA: NSO and ICF Macro, 2011.

[8] O’Shea MS, Rosenberg NE, Hosseinipour MC (2015). Effect of HIV status on fertility desire and knowledge of long-acting reversible contraception of postpartum Malawian women. AIDS Care.

[9] Damle LF, Gohari AC, McEvoy AK (2015). Early initiation of postpartum contraception: does it decrease rapid repeat pregnancy in adolescents?. J Pediatr Adolesc Gynecol.

[10] Hathaway M, Torres L, Vollett-Krech J (2014). Increasing LARC utilization: any woman, any place, any time. Clin Obstet Gynecol.

[11] Sober S, Schreiber CA (2014). Postpartum contraception. Clin Obstet Gynecol.

[12] Lopez LM, Grey TW, Chen M (2014). Strategies for improving postpartum contraceptive use: evidence from non-randomized studies. Cochrane Database Syst Rev.

[13] Zapata LB, Murtaza S, Whiteman MK (2015). Contraceptive counseling and postpartum contraceptive use. Am J Obstet Gynecol.

[14] Tang JH, Dominik R, Re S (2013). Characteristics associated with interest in long-acting reversible contraception in a postpartum population. Contraception.

[15] Anguzu R, Tweheyo R, Sekandi JN (2014). Knowledge and attitudes towards use of long acting reversible contraceptives among women of reproductive age in Lubaga division, Kampala district, Uganda. BMC Res Notes.

[16] Tilahun T, Coene G, Luchters S (2013). Family planning knowledge, attitude and practice among married couples in Jimma Zone, Ethiopia. PLoS One.

[17] Dhont N, Ndayisaba GF, Peltier CA (2009). Improved access increases postpartum uptake of contraceptive implants among HIV-positive women in Rwanda. Eur J Contracept Reprod Health Care.

[18] Haddad LB, Feldacker C, Jamieson DJ (2014). Medical eligibility, contraceptive choice, and intrauterine device acceptance among HIV-infected women receiving antiretroviral therapy in Lilongwe, Malawi. Int J Gynaecol Obstet.

[19] Phiri S, Feldacker C, Chaweza T, et al. Integrating reproductive health services into HIV care: strategies for successful implementation in a low-resource HIV clinic in Lilongwe, Malawi. *J Fam Plann Reprod Health Care* Published Online First: 22 April 2015. doi:10.1136/jfprhc-2013-100816. 10.1136/jfprhc-2013-100816 PMC4717379 25902815

[20] O’Shea M, Mwafulirwa T, Hamela G (2014). Family planning providers’ experiences and perceptions of long-acting reversible contraception in Lilongwe, Malawi [abstract]. Contraception.

[21] Crede S, Hoke T, Constant D (2012). Factors impacting knowledge and use of long acting and permanent contraceptive methods by postpartum HIV positive and negative women in Cape Town, South Africa: a cross-sectional study. BMC Public Health.

[22] Dassah ET, Odoi AT, Owusu-Asubonteng G (2013). Prevalence and factors predictive of long-acting reversible contraceptive use in a tertiary hospital in urban Ghana. Eur J Contracept Repro Health Care.

[23] Khu NH, Vwalika B, Karita E (2013). Fertility goal-based counseling increases contraceptive implant and IUD use in HIV-discordant couples in Rwanda and Zambia. Contraception.

[24] Tang JH, Dominik RC, Zerden ML (2014). Effect of an educational script on postpartum contraceptive use: a randomized controlled trial. Contraception.

[25] Dahlke JD, Ramseyer AM, Terpstra ER (2012). Postpartum use of long-acting reversible contraception in a military treatment facility. J Womens Health (Larchmt).

[26] Bolam A, Manandhar DS, Shrestha P (1998). The effects of postnatal health education for mothers on infant care and family planning practices in Nepal: a randomised controlled trial. BMJ.

[27] Saeed GA, Fakhar S, Rahim F (2008). Change in trend of contraceptive uptake--effect of educational leaflets and counseling. Contraception.

[28] Sarnquist CC, Moyo P, Stranix-Chibanda L (2014). Integrating family planning and prevention of mother to child HIV transmission in Zimbabwe. Contraception.

[29] McClain Burke H, Ambasa-Shisanya C (2014). Evaluation of a communication campaign to improve continuation among first-time injectable contraceptive users in Nyando District, Kenya. Int Perspect Sex Reprod Health.

[30] Purdy CH (2011). Using the Internet and social media to promote condom use in Turkey. Reprod Health Matters.

[31] Eke AC, Alabi-Isama L (2011). Long-acting reversible contraception (LARC) use among adolescent females in secondary institutions in Nnewi, Nigeria. J Obstet Gynaecol.

[32] Somba MJ, Mbonile M, Obure J (2014). Sexual behaviour, contraceptive knowledge and use among female undergraduates’ students of Muhimbili and Dar es Salaam Universities, Tanzania: a cross-sectional study. BMC Womens Health.

